# Cognitive and Neurochemical Effects of Brivaracetam Co-Administered with Ethanol: A Preclinical Study

**DOI:** 10.3390/ijms27167435

**Published:** 2026-08-20

**Authors:** Ewa Zwierzyńska, Joanna Stragierowicz, Marzenna Nasiadek, Michał Klimczak, Bogusława Pietrzak

**Affiliations:** 1 Department of Pharmacodynamics, Medical University of Lodz, Muszyńskiego 1, 90-151 Lodz, Poland; boguslawa.pietrzak@umed.lodz.pl; 2 Department of Toxicology, Medical University of Lodz, Muszyńskiego 1, 90-151 Lodz, Poland; joanna.stragierowicz@umed.lodz.pl (J.S.); marzenna.nasiadek@umed.lodz.pl (M.N.); michal.klimczak@umed.lodz.pl (M.K.)

**Keywords:** brivaracetam, ethanol, memory, neurotransmitter, oxidative stress, trace elements

## Abstract

Ethanol affects the central nervous system, with frequent use leading to memory impairment. This study aims to determine the effects of brivaracetam on ethanol-induced memory impairment and alterations in selected neurotransmitter levels, trace elements in the brain and serum oxidative stress parameters in rats. Brivaracetam was administered for three weeks (6 mg/kg b.w.) before behavioral tests. Ethanol was given as a forced model (5 g/kg b.w.). Spatial memory was evaluated with the Morris water maze test and recognition memory with the novel object recognition test. GABA and glutamate concentrations were evaluated in the cerebellum, hippocampus, and cerebral cortex, while superoxide dismutase (SOD) and catalase (CAT) activities and malondialdehyde (MDA) levels were measured in blood serum. The levels of copper, manganese, zinc, iron, and magnesium in the brain were also determined. Brivaracetam was found to negatively affect spatial and retrieval memory when administered with ethanol and during withdrawal; however, the drug improved ethanol-induced short-term recognition memory impairment. Furthermore, brivaracetam increased CAT activity, while MDA was decreased and SOD increased in all drug-treated animals. Brivaracetam may counteract ethanol-induced manganese brain concentration, but increases iron, magnesium, and copper concentrations. Hence, brivaracetam use induces various effects on the central nervous system.

## 1. Introduction

Brivaracetam ((2S)-2-[(4R)-2-oxo-4-propylpyrrolidin-1-yl]butanamide) is a novel antiseizure drug that binds to synaptic vesicle protein 2A (SV2A) in a similar way to levetiracetam. However, compared to levetiracetam, it demonstrates approximately 20-fold greater affinity for SV2A as a ligand [1], which may result from differences in the binding site or conformational state of the SV2A protein [2]. Also, while the binding sites of these drugs appear to be closely related, the two drugs exhibit different interactions [3]. A recent structural study of the SV2A–brivaracetam complex found that allosteric modulation of SV2A can influence the affinity of brivaracetam to this target and its stability [4].

SV2A is the most widely expressed SV2 protein across various brain regions, including the hippocampus, cerebellum, cortex, and subcortical areas including the thalamus and basal ganglia [5]. While its role has not yet been precisely established, it is known to be expressed in both γ-aminobutyric acid GABAergic and glutamatergic neuronal terminals and, as such, may modulate the balance between excitatory and inhibitory signals [6]. It is believed that SV2A takes part in calcium-dependent exocytosis, neurotransmitter retention in synaptic vesicles, synaptic vesicle priming, and transport of vesicle constituents [5]. In vitro, brivaracetam has been found to suppress astroglial glutamate release during seizures by either blocking aberrant SV2A expression or inhibiting Cx43 hemichannels, which are responsible for excessive glutamate release [7]. However, the drug did not appear to have a direct effect on ionotropic glutamate, GABA, or glycine receptors in hippocampal neurons [8].

Antiseizure drugs are known to have concerning adverse effects on cognitive functions, and while brivaracetam seems to have a favorable profile, available data are inconclusive. In our previous study, the drug administered for 14 days at a dose of 6 mg/kg body weight (b.w.) was associated with transient spatial and retrieval memory deficits in healthy rats [9]. Detrait et al. (2010) demonstrated that brivaracetam did not affect spatial and retrieval memory in either normal or amygdala-kindled rats after a single dose of brivaracetam 2.1, 6.8 or 21 mg/kg b.w. intraperitoneally (i.p.) [10]. In another study in rats with status epilepticus induced by kainic acid, as well as sham animals, brivaracetam administered at a single dose of 30 mg/kg i.p. did not affect spatial memory; however, retention memory was disturbed [11]. Moreover, brivaracetam given for 28 days at a dose of 8.5 mg/kg b.w. i.p. reduced spike-wave discharges and reversed spatial memory dysfunction in an Alzheimer’s disease mouse model [12]. These differences may be attributed to variations in experimental models, treatment protocols, and neuronal status.

Ethanol is known to have a complex effect on the central nervous system, and chronic exposure leads to memory impairment. This has been attributed to several mechanisms, including the disruption of glutamatergic and GABAergic neurotransmission, which are crucial processes in memory formation and retrieval. In preclinical studies, these cognitive deficits concern not only spatial memory but also working memory, contextual fear memory, and novel object recognition memory. Moreover, such dysregulation can also lead to decreased attention, mood changes, and consequently, to alcohol dependence [13,14]. Additionally, alcohol consumption induces neuroinflammation and oxidative stress, which contribute to the development of neurodegenerative processes in various regions of the brain; most notably, they affect the hippocampus, related to memory consolidation, and the prefrontal cortex, associated with executive functions. The induction of oxidative stress by ethanol may be mediated by various mechanisms, including free radical production, lipid peroxidation, crosslink formation and DNA strand breaks. Additionally, it can impair the activity of antioxidative enzymes including superoxide dismutase (SOD), catalase (CAT) and glutathione peroxidases (GPx) [13,15]. Moreover, alcohol dependence has been found to alter the levels of certain trace elements in the brain; among these, iron and copper influence the level of oxidative stress and neurodegeneration, while zinc is one of the essential elements in the antioxidant defense system. The presence of such changes can therefore reflect disturbances in oxidative stress pathways [16].

Brivaracetam has previously been observed to have a neuroprotective effect in a temporal lobe epilepsy model in rats [17]. While the drug has been found to influence the activity of antioxidant-related enzymes such as SOD and GPx in mice treated with pentylenetetrazole, more pronounced neuroprotective effects were observed when given in combination with perampanel [18]. However, unlike previous studies, the present study analyzes the impact of the drug on cognition associated with biochemical changes in animals exposed to ethanol. Previous research in this area has been limited to healthy volunteers, in whom brivaracetam was found to significantly increase the effects of ethanol on psychomotor function, attention and memory; the authors of this study do not recommend consuming brivaracetam with alcohol [19]. Generally, alcohol use is contraindicated with any antiepileptic drug; however, as many as 58–65% of patients with epilepsy are reported to have consumed alcohol in the past 12 months [20] and the prevalence of alcohol use disorder among patients with epilepsy ranges from 12 to 35% [21]. In response, the present study aims to enrich the body of experimental data regarding the effects of brivaracetam in the context of chronic ethanol exposure and withdrawal.

The study, therefore, examines the impact of brivaracetam, co-administered with ethanol, on spatial and associative memory, and the concentrations of GABA and glutamate in the cerebellum, hippocampus and cerebral cortex; it also examines its effect on selected oxidative stress parameters in the blood serum, viz. SOD, CAT activities and malondialdehyde (MDA) levels, and the concentration of selected essential elements, viz. copper, manganese, zinc, iron and magnesium, in brain tissue.

## 2. Results

### 2.1. Morris Water Maze Test (MWM)

#### 2.1.1. The Impact of Brivaracetam Administered with Ethanol on Spatial Memory in Rats

**Figure 1:**
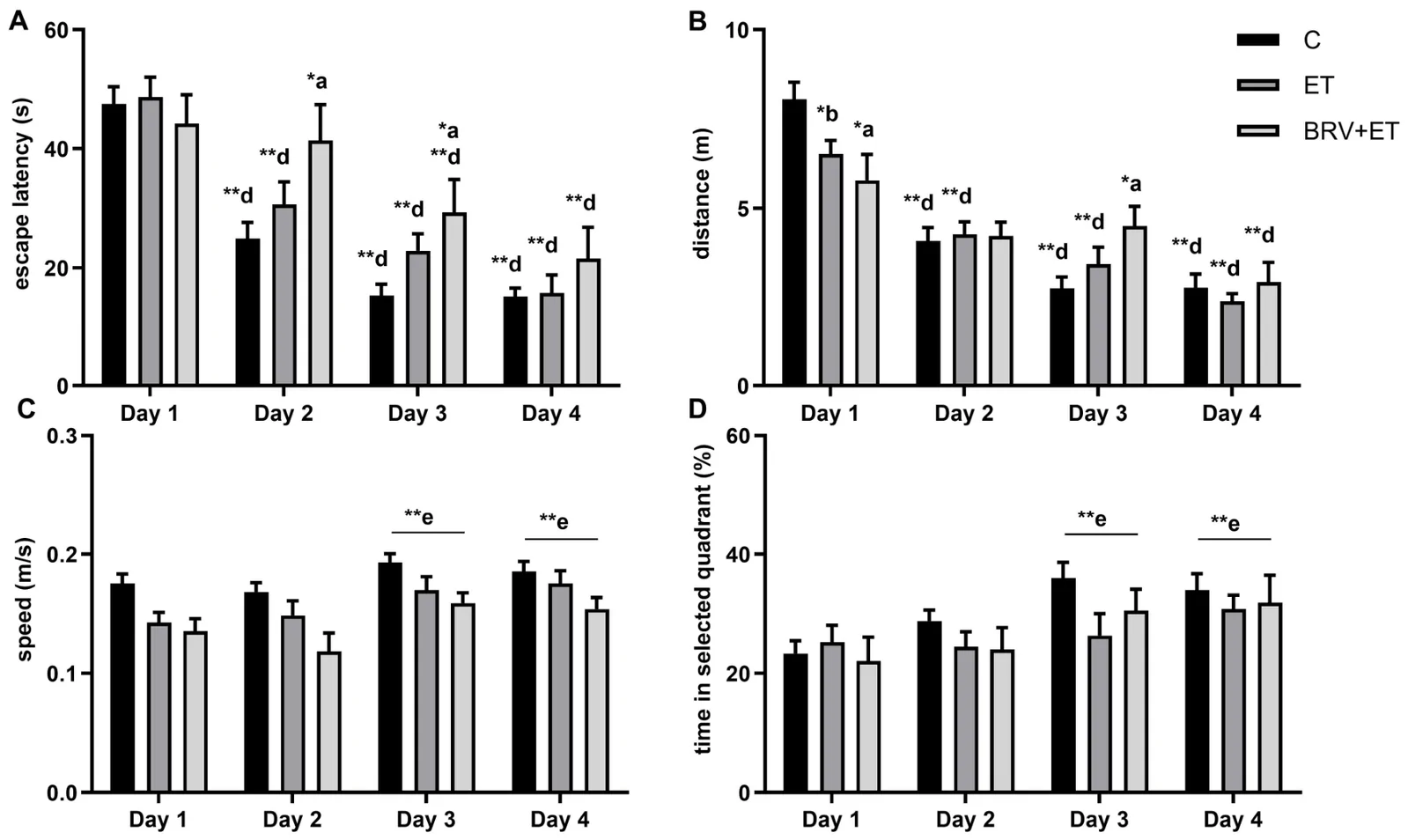
Effect of brivaracetam administered with ethanol on performance of rats in the MWM test: escape latency (**A**), distance (**B**), swimming speed (**C**), percentage of time spent in the selected quadrant (**D**) (*n* = 8). * *p* < 0.05, ** *p* < 0.01, two-way RM ANOVA with Tukey’s multiple comparisons test. a—significant difference between the brivaracetam and ethanol group (BRV + ET) and the control group (C) on that day, b—significant difference between the ethanol group (ET) and the control group (C) on that day, d—significant difference between particular test day and test day 1 (initial values), e—significant difference between particular test day and test day 1 (initial values) based on data from all animals.

Two-way RM ANOVA found both time × treatment interaction and time (F_(6,63)_ = 2.31, *p* = 0.0443, ηp^2^ = 0.18; F_(3,63)_ = 52.67, *p* < 0.0001, ηp^2^ = 0.71, respectively) to have a significant effect on escape latency during the four-day swimming trials. In Figure 1A, significant differences in escape latencies were found between the animals receiving brivaracetam with ethanol (BRV + ET) and the control group (C) on the second day (*p* = 0.0103) and on the third day (*p* = 0.0329). In all groups, the escape latencies were significantly reduced on days 3 and 4 compared to initial values (C: Day 3 *p* < 0.0001, Day 4 *p* < 0.0001; ET: Day 3 *p* < 0.0001, Day 4 *p* < 0.0001; BRV + ET: Day 3 *p* = 0.0060, Day 4 *p* < 0.0001, respectively); it was also significantly lower on Day 2 in the C (*p* < 0.0001) and ET groups (*p* = 0.0006). Two-way RM ANOVA analyses for the distance traveled showed that both the time × treatment interaction and time had significant effects (F_(6,63)_ = 3.54, *p* = 0.0044, ηp^2^ = 0.25; F_(3,63)_ = 46.25, *p* < 0.0001, ηp^2^ = 0.69, respectively) (Figure 1B).

Similar to escape latency, the BRV + ET group demonstrated increased distance traveled on Day 3 compared to the C group (*p* = 0.0203). However, on Day 1, the C group swam significantly longer compared to the BRV + ET (*p* = 0.0017) and ET groups (*p* = 0.0478). In all groups, traveled distance was shorter on the last day compared to initial values (*p* < 0.0001 in all groups); the distance was also shorter on Days 2 and 3 in C and ET groups (C: Day 2 *p* < 0.0001, Day 3 *p* < 0.0001; ET: Day 2 *p* = 0.0041, Day 3 *p* < 0.0001, respectively).

Swimming speed was affected by time (F_(3,63)_ = 14.91, *p* < 0.0001, ηp^2^ = 0.42), but no effect was observed for the treatment × time interaction (Figure 1C). Animals from all three groups swam faster on Day 3 (*p* = 0.0003) and Day 4 (*p* = 0.0015) compared to Day 1. Also, a similar time effect was noted for percentage time in the target quadrant (F_(3,63)_ = 7.46, *p* = 0.0002, ηp^2^ = 0.26): all animals spent more time in the target quadrant with the platform on Days 3 (*p* = 0.0055) and 4 (*p* = 0.0008) compared to Day 1 (Figure 1D).

**Figure 2:**
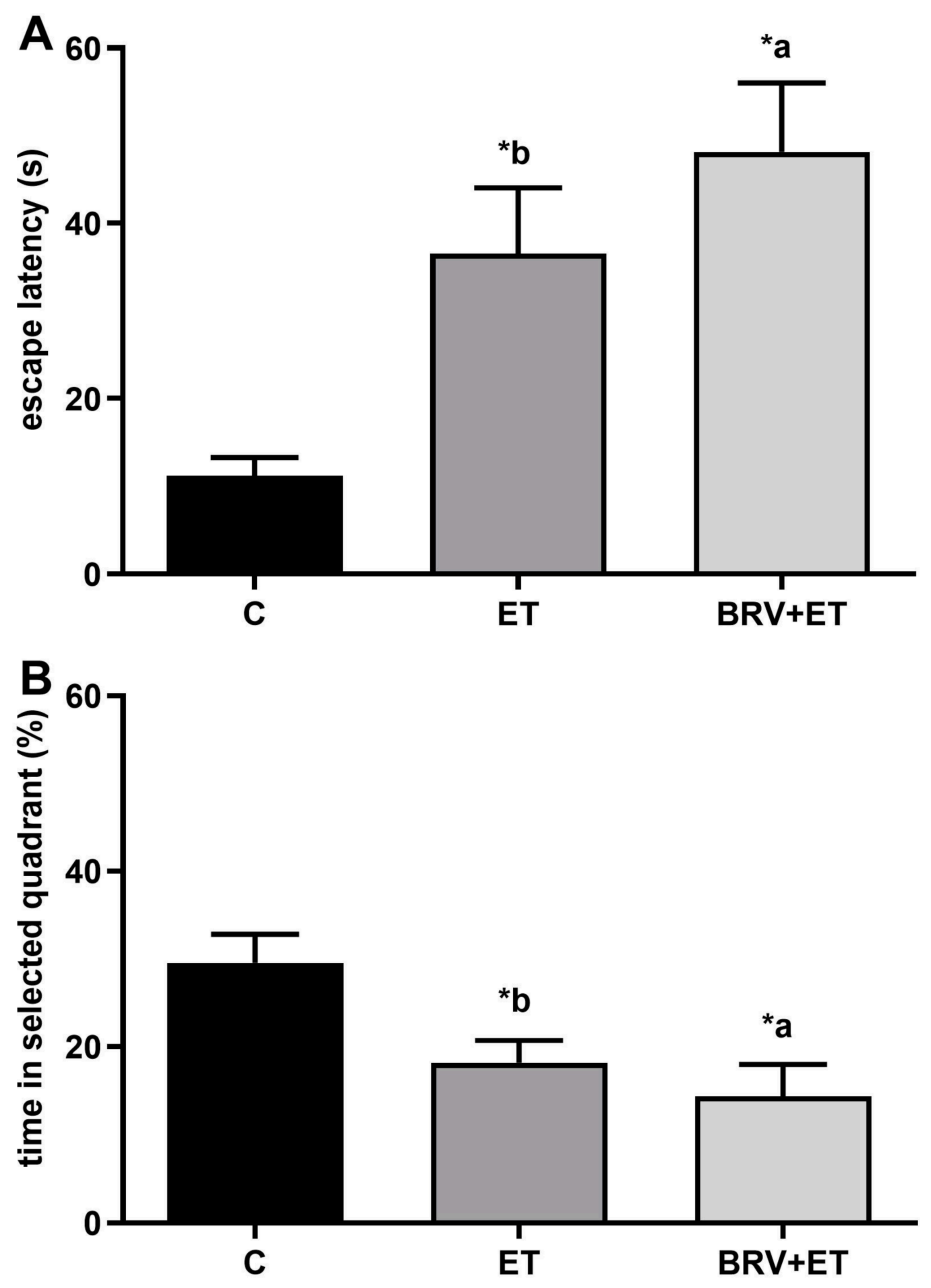
Effect of brivaracetam administered with ethanol on the performance of rats on the probe day (fifth day) of the MWM test: time needed to identify the previous location of the platform (**A**), the time spent in the zone where the platform was located (**B**) (*n* = 8). * *p* < 0.05, one-way ANOVA with Sidak’s multiple comparisons test. a—significant difference between the BRV + ET group and the C group; b—significant difference between the ET group and the Cgroup.

On the probe day, one-way ANOVA showed significant differences between groups in escape latency (F_(2,21)_ = 8.76, *p* = 0.0017, ηp^2^ = 0.46). The ET and BRV + ET groups would find the platform in a longer time than the C group (ET: *p* = 0.0273; BRV + ET: *p* = 0.0016) (Figure 2A). Moreover, a significant effect was also observed in the percentage of time in the quadrant (F_(2,21)_ = 6.23, *p* = 0.0075, ηp^2^ = 0.37). Both ET and BRV + ET groups spent a smaller percentage of time in the quadrant where the platform had previously been located (ET: *p* = 0.0478; BRV + ET: *p* = 0.0075) (Figure 2B).

#### 2.1.2. The Impact of Brivaracetam on the Spatial Memory in Rats 24 h from Discontinuation of Ethanol Administration

**Figure 3:**
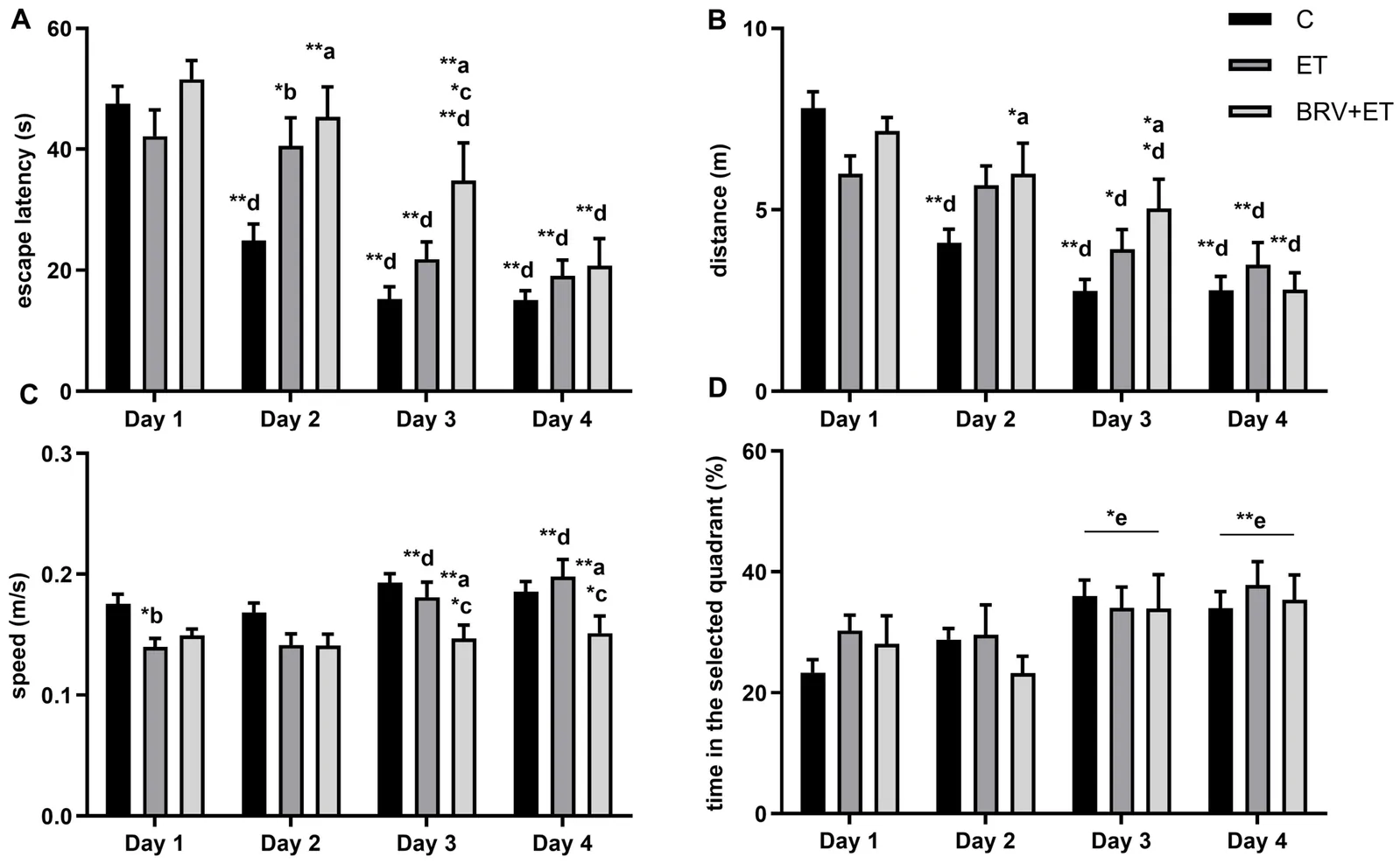
Effect of brivaracetam on performance of rats in the MWM test 24 h after discontinuation of ethanol administration: escape latency (**A**), distance (**B**), swimming speed (**C**), percentage of time spent in the selected quadrant (**D**) (*n* = 8). * *p* < 0.05, ** *p* < 0.01, two-way RM ANOVA with Tukey’s multiple comparisons test. a—significant difference between the BRV + ET group and the C group on that day; b—significant difference between the ET group and the C group on that day; c—significant difference between the BRV + ET group and the ETl group on that day; d—significant difference between particular test day and test day 1 (initial values); e—significant difference between particular test day and test day 1 (initial values) based on data from all animals.

Two-way RM ANOVA revealed that time (F_(3,63)_ = 51.43, *p* < 0.0001, ηp^2^ = 0.71), treatment (F_(2,21)_ = 5.64, *p* = 0.0109, ηp^2^ = 0.35) and the treatment × time interaction (F_(6,63)_ = 3.00, *p* = 0.0122, ηp^2^ = 0.22) had effect on escape latency in rats (Figure 3A). On Day 2, escape latency was increased in the animals that had received previously only ethanol (ET group) compared to the C group (*p* = 0.0124). Rats from the BRV + ET group also demonstrated longer escape latency on Day 2 compared to the C group (*p* = 0.0007) and on Day 3 compared to both C and ET groups (*p* = 0.0012; *p* = 0.0444, respectively). However, the escape latency significantly decreased in all groups on Days 3 and 4 compared to Day 1 (C: *p* < 0.0001, *p* < 0.0001; ET: *p* = 0.0001, *p* < 0.0001; BRV + ET: *p* = 0.0019, *p* < 0.0001, respectively). In the C group, this time was also shortened on Day 2 compared to Day 1 (*p* < 0.0001).

Both the time × treatment interaction and time also had significant effects on the distance traveled by rats (F_(6,63)_ = 3.65, *p* = 0.0036, ηp^2^ = 0.26; F_(3,63)_ = 38.70, *p* < 0.0001, ηp^2^ = 0.65, respectively) (Figure 3B). Rats from the BRV + ET group traveled longer distances to find the platform on Days 2 and 3 compared to the C group (*p* = 0.0391; *p* = 0.0108, respectively). However, all animals showed reduced distance with subsequent days, and significant differences were observed in all groups between Days 1 and 3 as well as between Days 1 and 4 (C: *p* < 0.0001, *p* < 0.0001; ET: *p* = 0.0167, *p* = 0.0026; BRV + ET: *p* = 0.0134, *p* < 0.0001, respectively). This decrease was also significant in the C group on Day 2 compared to the initial values (*p* < 0.0001).

Two-way RM ANOVA also indicated that swimming speed was affected by time (F_(3,63)_ = 11.75, *p* < 0.0001, ηp^2^ = 0.36), treatment (F_(2,21)_ = 4.42, *p* = 0.0251, ηp^2^ = 0.30) and their interaction (F_(6,63)_ = 4.36, *p* = 0.0010, ηp^2^ = 0.29) (Figure 3C). On Day 1, the rats from the ET group demonstrated the lowest swimming speed; this value was significantly different from that of the C group (*p* = 0.036). However, as the test progressed, the most pronounced decreases in speed were noted in the BRV + ET group, and this reduction was significant compared to both C and ET groups on Days 3 and 4 (C: Day 3 *p* = 0.0044, Day 4 *p* = 0.0438; ET: Day 3 *p* = 0.0467, Day 4 *p* = 0.0037, respectively). In the ET group, swimming speed increased on Days 3 (*p* = 0.0005) and 4 (*p* < 0.0001) compared to initial values.

Neither the treatment × time interaction nor treatment had any effect on percentage time spent by rats in the target quadrant; however, it was significantly affected only by time (F_(3,63)_ = 7.96, *p* = 0.0001, ηp^2^ = 0.27) (Figure 3D). On Days 3 and 4, all animals spent more time in the selected quadrant compared to Day 1 (*p* = 0.0114; *p* = 0.0028, respectively).

**Figure 4:**
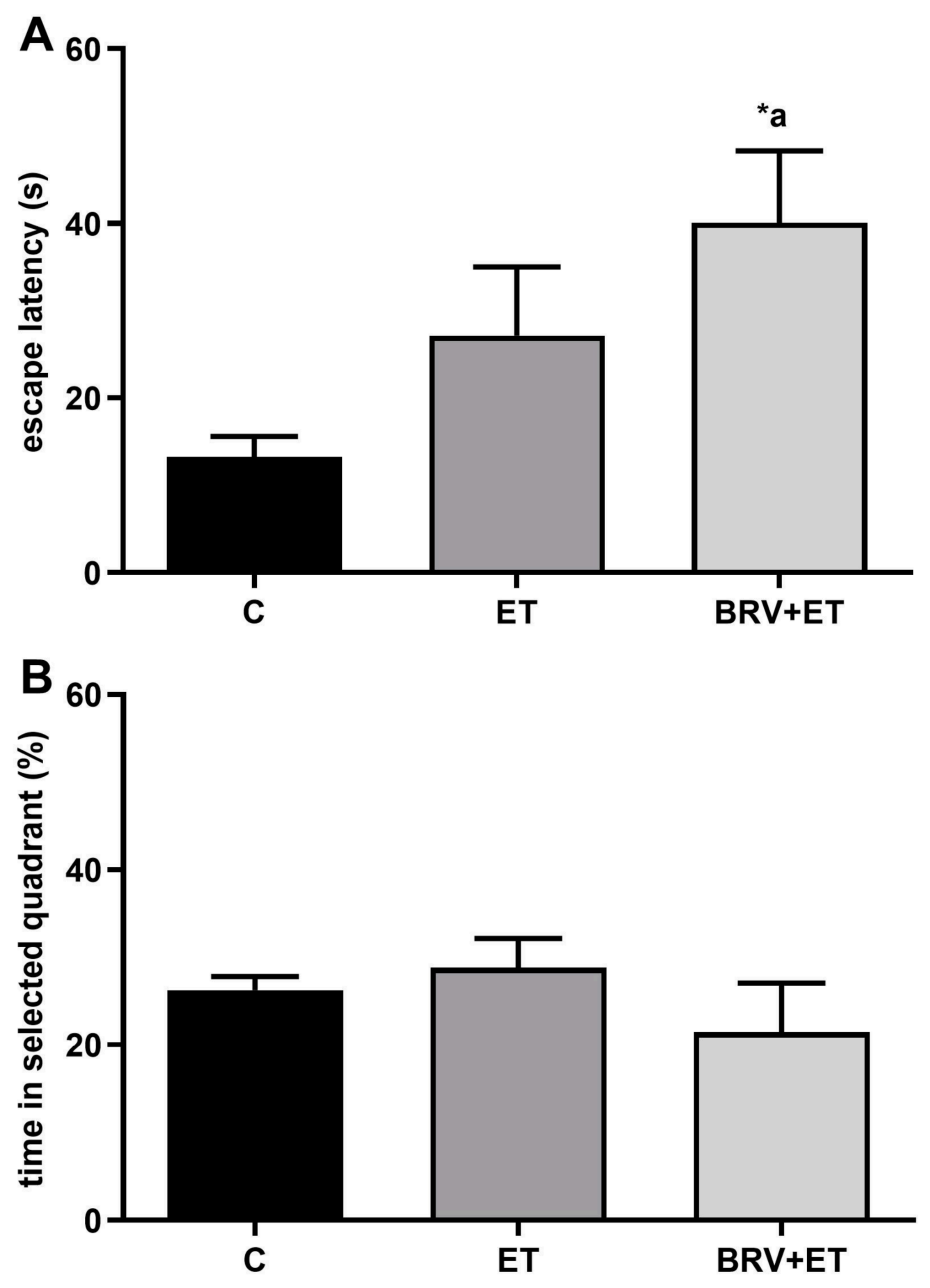
Effect of brivaracetam on the performance of rats on the probe day (fifth day) of the MWM test 24 h after discontinuation of ethanol administration: time needed to identify the previous location of the platform (**A**), the time spent in the zone where the platform was located (**B**) (*n* = 8). * *p* < 0.05, one-way ANOVA with Sidak’s multiple comparisons test. a—significant difference between the BRV + ET group and the C group.

On the probe day, one-way ANOVA showed a significant difference between groups in escape latency (F_(2,21)_ = 3.99, *p* = 0.0341, ηp^2^ = 0.28). The BRV + ET group would need more time to find the platform compared to the C group (*p* = 0.0264) (Figure 4A). No differences between groups were observed regarding the percentage of time spent in the quadrant where the platform had previously been located (Figure 4B).

### 2.2. Novel Object Recognition

#### The Impact of Brivaracetam Administered with Ethanol on Short-Term and Long-Term Recognition Memory in Rats

**Figure 5:**
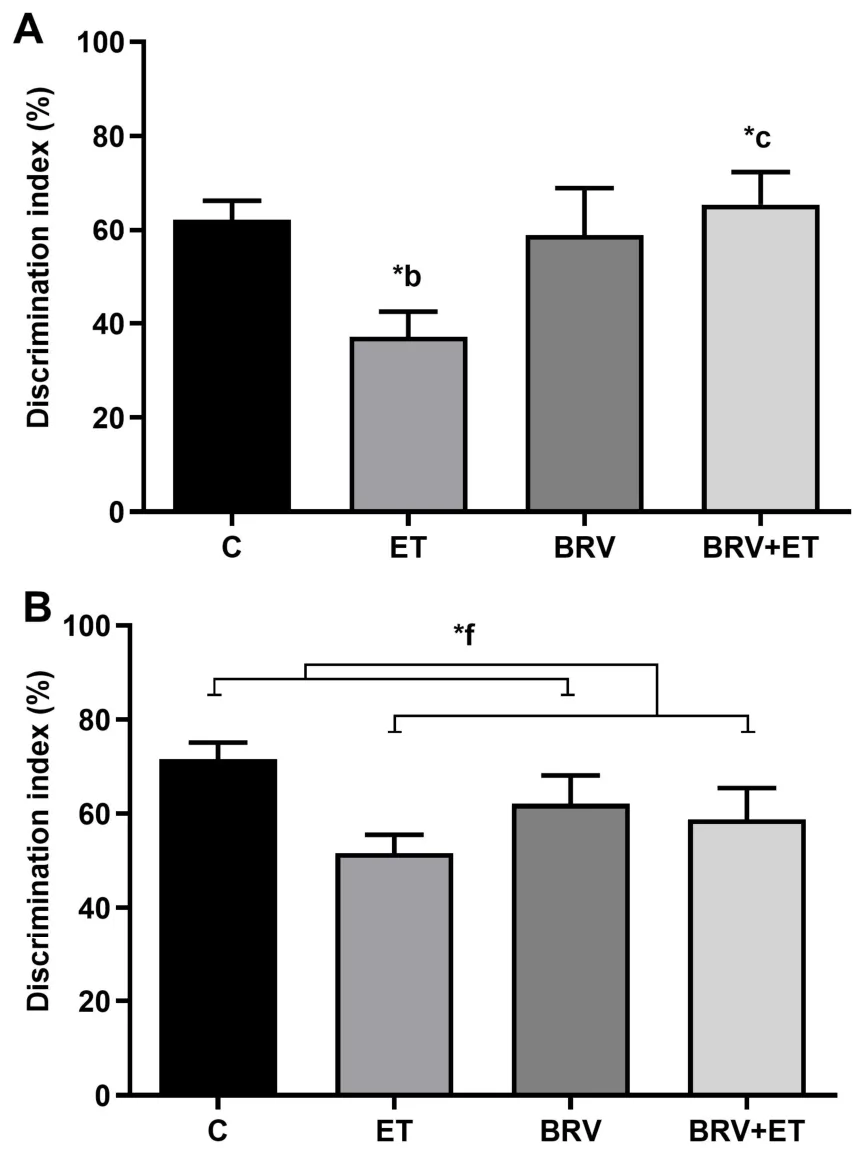
Effect of brivaracetam administered with ethanol on short-term (**A**) and long-term (**B**) recognition memory in rats. * *p* < 0.05, two-way ANOVA with Sidak’s multiple comparisons test (**A**) or two-way ANOVA with an unpaired *t*-test (**B**). b—significant difference between the ET group and the C group; c—significant difference between the BRV + ET group and the ET group; f—significant difference between ethanol-drinking rats (BRV + ET, ET) and non-drinking rats (C, BRV).

Two-way ANOVA revealed that the ethanol × drug interaction affected the discrimination index during short-term memory assessment (F_(1,28)_ = 5.14, *p* = 0.0313, ηp^2^ = 0.16) (Figure 5A). The ET group demonstrated a significantly lower discrimination index compared to the BRV + ET and C groups (*p* = 0.0162; *p* = 0.0338, respectively).

In the long-term memory test, only ethanol influenced the discrimination index (F_(1,28)_ = 5.01, *p* = 0.0334, ηp^2^ = 0.15) (Figure 5B). All animals receiving ethanol (ET and BRV + ET groups) demonstrated a shorter discrimination index than non-drinking rats (BRV and C groups) (*p* = 0.0345).

### 2.3. Blood Ethanol Level

No differences in blood ethanol level were observed between ET and BRV + ET groups (ET: 1.719 ± 0.187‰ versus BRV + ET: 1.475 ± 0.145‰) (figure not included).

### 2.4. Neurotransmitter Assay

#### 2.4.1. The Impact of Brivaracetam Administered with Ethanol on Glutamate and GABA in the Cerebellum (CB)

**Figure 6:**
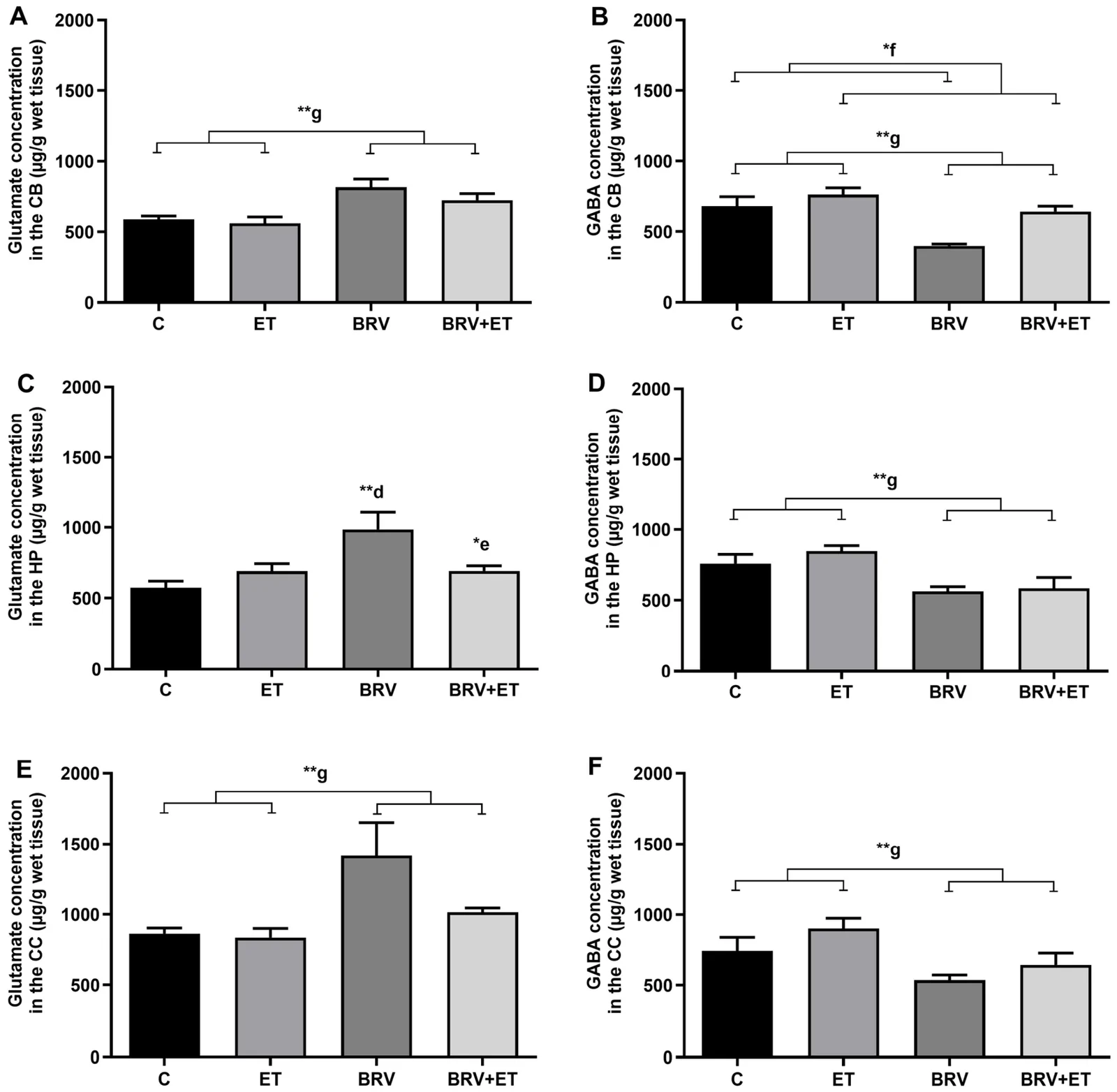
Effect of brivaracetam administered with ethanol on glutamate (**A**,**C**,**E**) and GABA levels (**B**,**D**,**F**) in the cerebellum (CB) (**A**,**B**), hippocampus (HP) (**C**,**D**), and cerebral cortex (CC) (**E**,**F**) (*n* = 8). * *p* < 0.05, ** *p* <  0.01, two-way ANOVA with an unpaired *t*-test (**A**,**B**,**D**–**F**) or two-way ANOVA with Sidak’s multiple comparisons test (**C**). d—significant difference between the brivaracetam group (BRV) and the C control group, e—significant difference between the BRV + ET group and the BRV group, f—significant difference between ethanol-drinking rats (BRV + ET, ET) and non-drinking rats (C, BRV), g—significant difference between brivaracetam-treated rats (BRV + ET, BRV) and non-drug rats (C, ET).

Two-way ANOVA revealed that neither the ethanol × drug interaction nor ethanol demonstrated any effect on glutamate level in the CB. However, the drug had a significant effect on glutamate (F_(1,28)_ = 18.98, *p* = 0.0002, ηp^2^ = 0.40) (Figure 6A): all animals receiving brivaracetam (BRV and BRV + ET groups) showed increased glutamate levels compared to non-drug rats (C and ET groups) (*p* = 0.0001). Moreover, the ethanol × drug interaction also had no effect on GABA concentration; however, the drug (F_(1,28)_ = 19.21; *p* = 0.0001, ηp^2^ = 0.41) and ethanol alone (F(_1,28)_ = 12.62; *p* = 0.0014, ηp^2^ = 0.31) exhibited an effect (Figure 6B). All rats receiving brivaracetam (BRV and BRV + ET groups) showed decreased GABA levels compared to non-drug animals (C and ET groups) (*p* = 0.0010). Furthermore, ethanol-drinking rats (ET and BRV + ET groups) demonstrated higher GABA levels than non-drinking animals (BRV and C groups) (*p* = 0.0101).

#### 2.4.2. The Impact of Brivaracetam Administered with Ethanol on Glutamate and GABA in the Hippocampus (HP)

Two-way ANOVA showed that ethanol × drug interaction affects the HP glutamate level (F_(1,28)_ = 7.92; *p* = 0.0089, ηp^2^ = 0.22) (Figure 6C). Brivaracetam significantly increased the level of glutamate compared to C (*p* = 0.0008) and BRV + ET groups (*p* = 0.0163). In addition, neither the drug × ethanol interaction nor ethanol had any effect on HP GABA concentration; however, the drug had an effect (F_(1,28)_ = 16.75, *p* = 0.0003, ηp^2^ = 0.37) (Figure 6D). Animals from the BRV and BRV + ET groups demonstrated a decreased GABA level compared to all non-drug groups (ET and C groups) (*p* = 0.0003).

#### 2.4.3. The Impact of Brivaracetam Administered with Ethanol on Glutamate and GABA in the Cerebral Cortex (CC)

Neither the drug × ethanol interaction nor ethanol treatment demonstrated any effects on CC GABA or glutamate concentration; however, the drug exhibited an effect on both glutamate (F_(1,28)_ = 8.64; *p* = 0.0065, ηp^2^ = 0.24) and GABA (F_(1,28)_ = 9.20; *p* = 0.0052, ηp^2^ = 0.25). Brivaracetam in animals from the BRV and BRV + ET groups increased glutamate concentration (*p* = 0.0090) (Figure 6E), but decreased GABA compared to the non-drug groups (ET and C groups) (*p* = 0.0058) (Figure 6F).

### 2.5. Serum Oxidative Stress Markers

#### The Impact of Brivaracetam Administered with Ethanol on MDA, SOD and CAT Activity in Rat Blood

**Figure 7:**
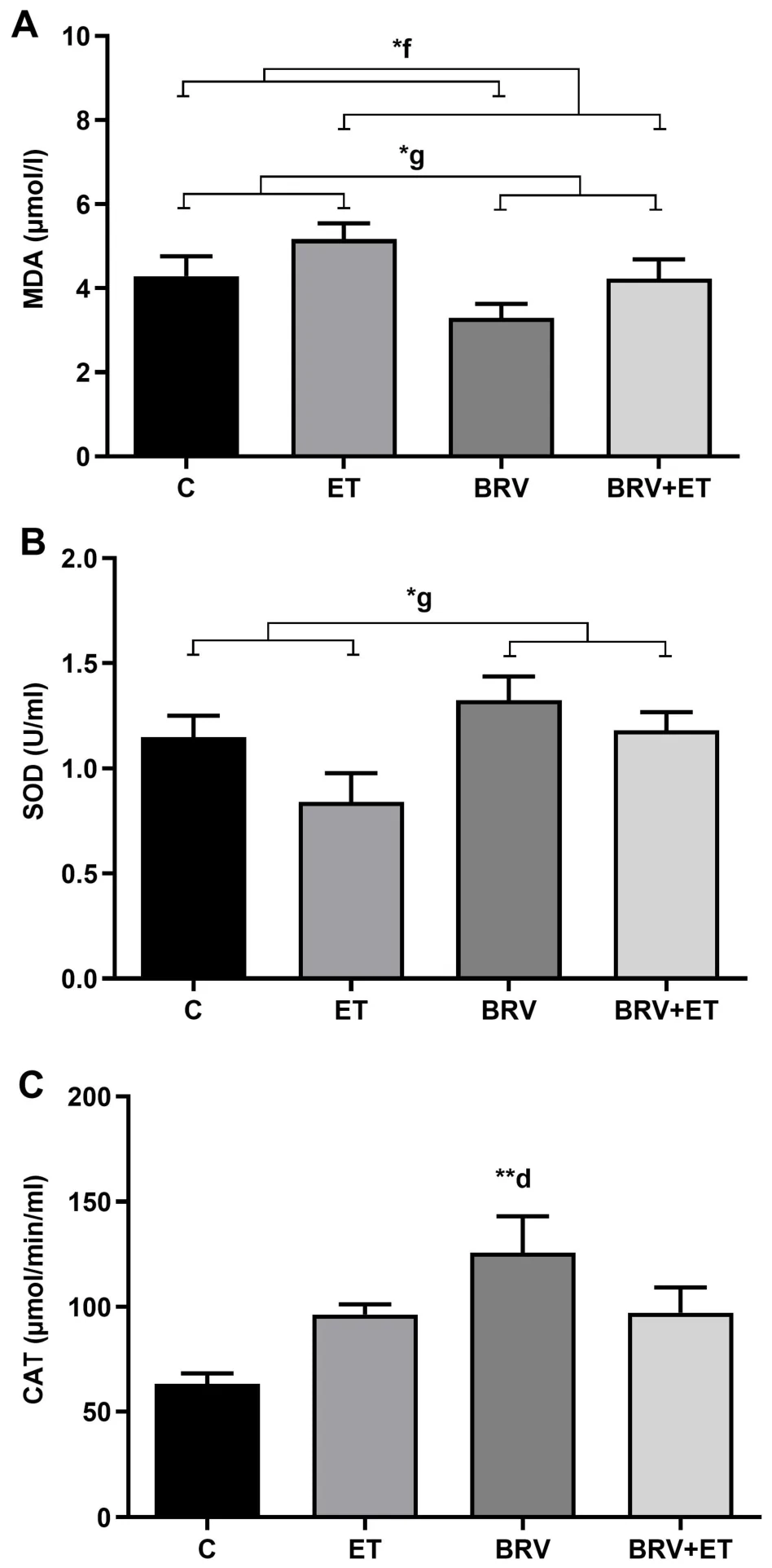
Effect of brivaracetam administered with ethanol on MDA (**A**), SOD (**B**), and CAT (**C**) in the serum of rats (*n* = 8). * *p* < 0.05, ** *p* <  0.01, two-way ANOVA with an unpaired *t*-test (**A**,**B**) or two-way ANOVA with Sidak’s multiple comparisons test (**C**). d—significant difference between the BRV group and the C c group, f—significant difference between ethanol-drinking rats (BRV + ET, ET) and non-drinking rats (C, BRV), g—significant difference between brivaracetam-treated rats (BRV + ET, BRV) and non-drug rats (C, ET).

Two-way ANOVA revealed that ethanol (F_(1,28)_ = 4.83; *p* = 0.0365, ηp^2^ = 0.15) and the drug (F_(1,28)_ = 5.48; *p* = 0.0266, ηp^2^ = 0.16) were found to affect MDA level; however, drug × ethanol interaction had any effect (Figure 7A). All animals receiving ethanol (ET and BRV + ET groups) showed increased MDA levels compared to all non-alcoholic rats (BRV and C groups) (*p* = 0.0462). Additionally, animals from the BRV and BRV + ET groups demonstrated decreased MDA levels compared to non-drug rats (ET and C groups) (*p* = 0.0328).

Neither the drug × ethanol interaction nor ethanol had any effects on SOD activity. However, an effect was observed for the drug (F_(1,28)_ = 5.38; *p* = 0.0279, ηp^2^ = 0.16) (Figure 7B); all animals receiving the drug (BRV and BRV + ET groups) demonstrated higher SOD levels than non-drug rats (ET and C groups) (*p* = 0.0340). Furthermore, the drug × ethanol interaction (F_(1,28)_ = 7.89, *p* = 0.0090, ηp^2^ = 0.22) and the drug (F_(1,28)_ = 8.30; *p* = 0.0075, ηp^2^ = 0.23) were found to have effects on CAT activity (Figure 7C). Brivaracetam significantly increased CAT activity compared to controls (*p* = 0.0008).

### 2.6. The Influence on Essential Elements Concentrations in the Brain

#### The Impact of Brivaracetam Administered with Ethanol on Selected Essential Elements Concentrations in the Brain

**Figure 8:**
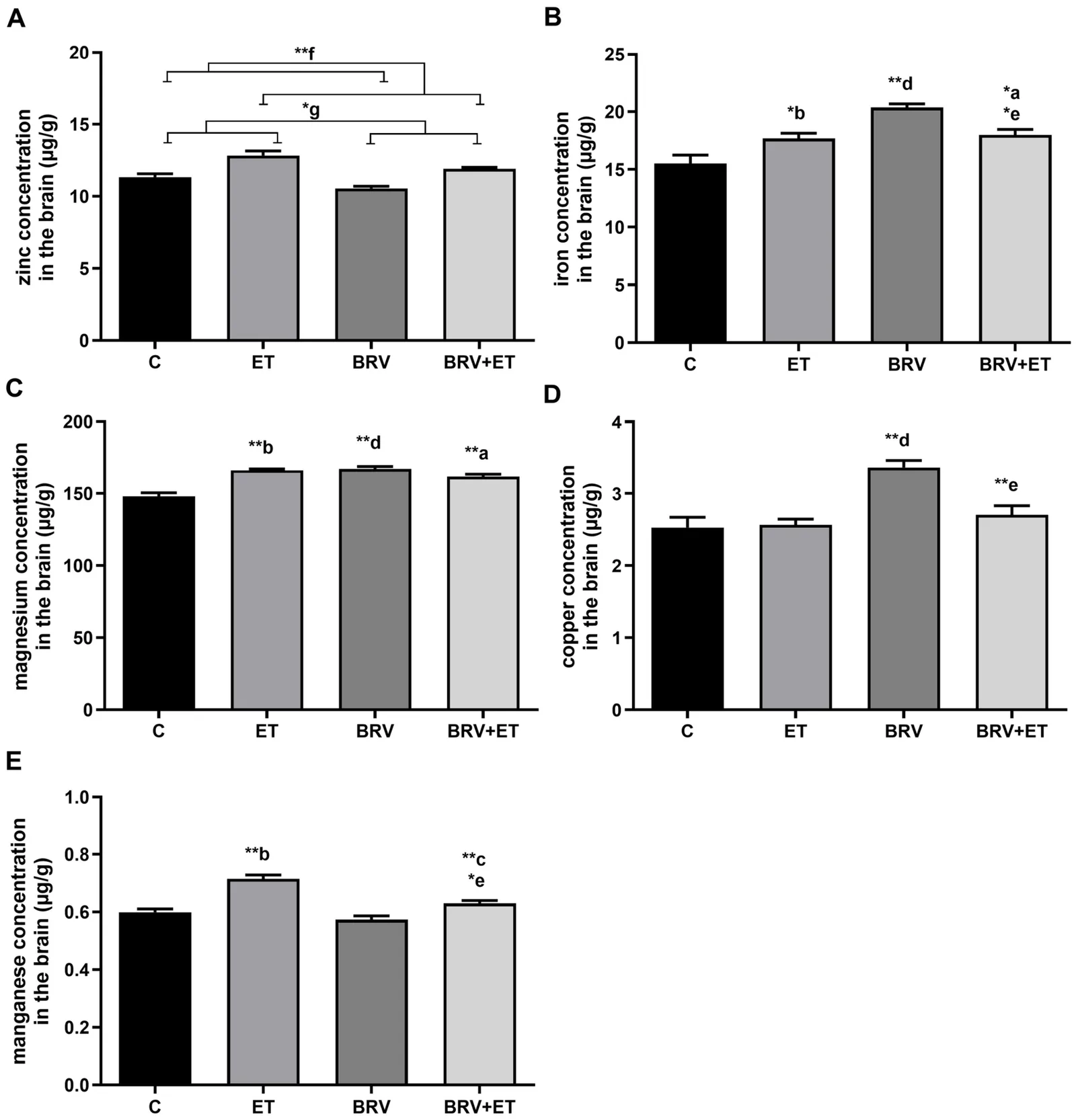
Effect of brivaracetam administered with ethanol on zinc (**A**), iron (**B**), magnesium (**C**), copper (**D**) and manganese concentration (**E**) in the brain of rats. * *p* < 0.05, ** *p* <  0.01, two-way ANOVA with an unpaired *t*-test (**A**) or two-way ANOVA with Sidak’s multiple comparisons test (**B**–**E**). a—significant difference between the BRV + ET group and the C group, b—significant difference between the ET group and the C group (C), c—significant difference between the BRV + ET group and the C cgroup, d—significant difference between the BRV group and the C group (C), e—significant difference between the BRV + group and the BRVgroup), f—significant difference between ethanol-drinking rats (BRV + ET, ET) and non-drinking rats (C, BRV), g—significant difference between brivaracetam-treated rats (BRV + ET, BRV) and non-drug rats (C, ET).

Two-way ANOVA showed that ethanol (F_(1,28)_ = 44.76; *p* < 0.0001, ηp^2^ = 0.61) and drug (F_(1,28)_ = 15.69; *p* = 0.0005, ηp^2^ = 0.36) had significant effects on zinc concentration in the brain; no effect of the drug × ethanol interaction was observed (Figure 8A). All animals receiving ethanol (ET and BRV + ET groups) had higher levels of zinc compared to non-drinking rats (BRV and C groups) (*p* < 0.0001). Additionally, both groups receiving brivaracetam (BRV and BRV + ET groups) showed significantly lower zinc concentrations than the non-drug groups (ET and C groups) (*p* = 0.0164). The drug × ethanol interaction (F_(1,28)_ = 18.89; *p* = 0.0002, ηp^2^ = 0.40) and drug alone (F_(1,28)_ = 24.45; *p* < 0.0001, ηp^2^ = 0.47) had a significant effect on iron concentration (Figure 8B). The highest iron level was noted in the BRV group, and these differences were significant compared to the BRV + ET (*p* = 0.0157) and C groups (*p* < 0.0001). A significant difference was also noted between the BRV + ET and C groups (*p* = 0.0121) as well as C and ET groups (*p* = 0.0330). The interaction, drug and ethanol alone had significant effects on magnesium (F_(1,28)_ = 45.10; *p* < 0.0001, ηp^2^ = 0.62; F_(1,28)_ = 16.83; *p* = 0.0003, ηp^2^ = 0.38; F_(1,28)_ = 12.04; *p* = 0.0017, ηp^2^ = 0.30, respectively). All three groups (ET, BRV and BRV + ET) demonstrated higher magnesium concentrations compared to the C group, with significant differences (*p* < 0.0001, all groups) (Figure 8C). The interaction between drug and ethanol had significant effects on copper (F_(1,28)_ = 9.23; *p* = 0.0051, ηp^2^ = 0.28; F_(1,28)_ = 18.02; *p* = 0.0002, ηp^2^ = 0.39; F_(1,28)_ = 7.40; *p* = 0.0111, ηp^2^ = 0.21, respectively). Significantly higher levels of copper were found in the BRV group compared to the BRV + ET and C groups (*p* = 0.0007; *p* < 0.0001, respectively) (Figure 8D). The interaction between drug and ethanol also had significant effects on manganese concentrations (F_(1,28)_ = 6.11; *p* = 0.0198, ηp^2^ = 0.18; F_(1,28)_ = 21.63; *p* < 0.0001, ηp^2^ = 0.44; F_(1,28)_ = 52.09; *p* < 0.0001, ηp^2^ = 0.65, respectively). Significantly higher levels of manganese were found in the ET group than either the BRV + ET or C group (*p* < 0.0001 for both). However, the level of manganese was higher in the BRV + ET group than in the BRV group (*p* = 0.0116) (Figure 8E).

### 2.7. The Correlation Between Changes in Discrimination Index and the Biochemical Parameters

**Figure 9:**
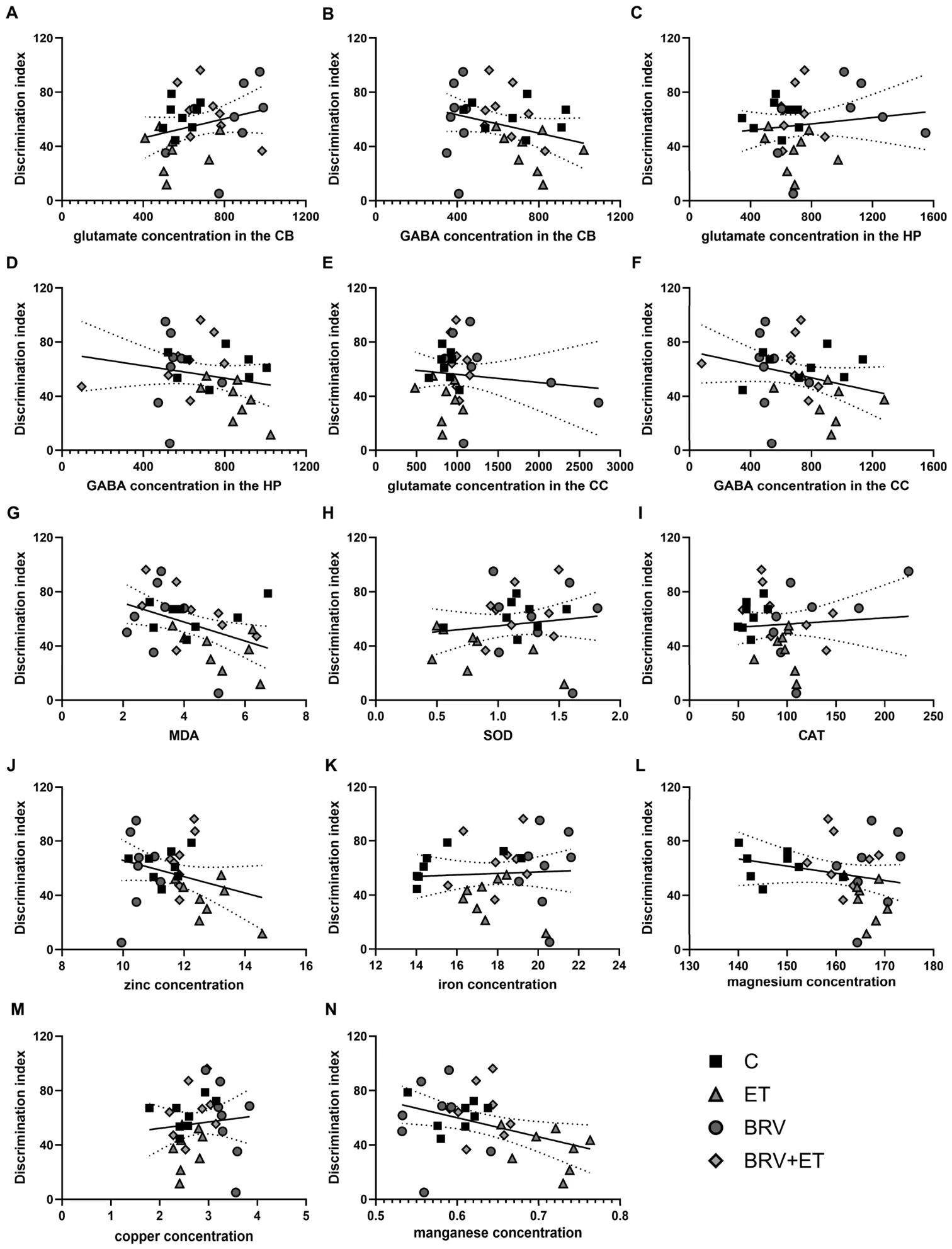
Pearson’s correlation of the discrimination index of short-term memory and glutamate in the CB (**A**), GABA in the CB (**B**), glutamate in the HP (**C**), GABA in the HP (**D**), glutamate in the CC (**E**), GABA in the CC (**F**), MDA (**G**), SOD (**H**), CAT (**I**), zinc (**J**), iron (**K**), magnesium (**L**), copper (**M**) and manganese (**N**).

Neither GABA nor glutamate concentrations were found to correlate with the discrimination index of short-term memory in any studied brain structure (Figure 9A–F). However, the discrimination index of short-term memory was negatively correlated with MDA (r = −0.4141; *p* = 0.0185, Figure 9G). No correlation was found with SOD (Figure 9H) or CAT (Figure 9I). The concentrations of zinc, iron, magnesium, and copper were not correlated with the discrimination index of short-term memory (Figure 9J–M). However, the manganese concentration was negatively correlated with the discrimination index of short-term memory (r = −0.4022; *p* = 0.0225, Figure 9N).

**Figure 10:**
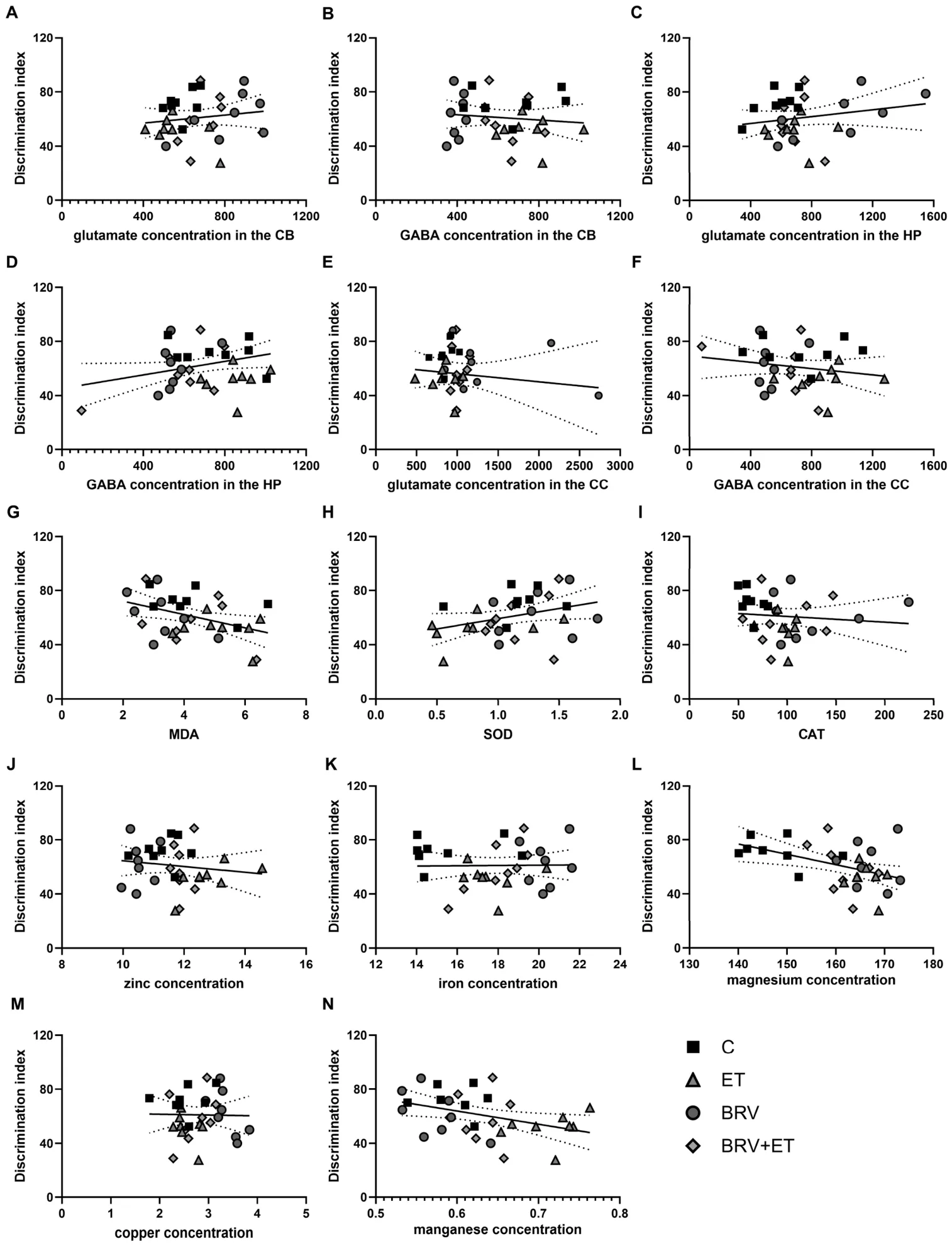
Pearson’s correlation of the discrimination index of long-term memory and glutamate in the CB (**A**), GABA in the CB (**B**), glutamate in the HP (**C**), GABA in the HP (**D**), glutamate in the CC (**E**), GABA in the CC (**F**), MDA (**G**), SOD (**H**), CAT (**I**), zinc (**J**), iron (**K**), magnesium (**L**), copper (**M**) and manganese (**N**).

Also, neither GABA nor glutamate concentration correlated with the discrimination index of long-term memory in any studied brain structure (Figure 10A–F). Similarly to short-term memory, the discrimination index of long-term memory was negatively correlated with MDA (r = −0.4044; *p* = 0.0217, Figure 10G). There was no correlation with SOD (Figure 10H) or CAT (Figure 10I). The concentrations of magnesium (Figure 10L) and manganese (Figure 10N) were negatively correlated with the discrimination index of long-term memory (r = −0.4442; *p* = 0.0109, r = −0.3867; *p* = 0.0288, respectively). No correlations were observed in relation to zinc (Figure 10J), iron (Figure 10K) or copper concentrations (Figure 10M).

## 3. Discussion

The present study investigated the effects of brivaracetam on ethanol-induced memory processes in rats and its impact on the levels of selected neurotransmitters, oxidative stress parameters, and essential elements. The effect of the drug on spatial memory when co-administered with ethanol or after a 24 h withdrawal period was determined with the MWM test. During the alcohol administration phase, brivaracetam temporarily disturbed memory, with particularly strong effects observed on days two and three, resulting in increased escape latency and distance traveled. However, these animals demonstrated improvements in these parameters over the last test days, suggesting that the learning process appeared to be only slowed. Alterations in retrieval memory were also noted in rats receiving brivaracetam and ethanol, and these were also pronounced in the ethanol group, as shown by an increase in potential escape latency and a decrease in time spent in the target quadrant. Similar temporary spatial memory disturbances were observed 24 h after discontinuation of ethanol administration. The rats treated with brivaracetam demonstrated longer escape latency and distance traveled on the second and third test days; they also indicated slower swimming speed on days three and four. The ethanol group indicated similar changes in swimming speed on the first test day and escape latency on the second test day. During withdrawal, the BRV + ET group also demonstrated retrieval memory disorders, similarly to when the drug was co-administered with ethanol.

This is the first study to assess the effect of brivaracetam on spatial memory in animals consuming ethanol; previous studies have only examined the effects of the drug when administered alone. Our previous research indicated that brivaracetam administered at a dose of 6 mg/kg b.w. i.g. for 14 days caused transient spatial memory deficits in rats; this negative effect was found to be dose- and time-dependent, as it was not observed when the drug was administered at single doses (6 mg/kg or 20 mg/kg b.w.). Moreover, retrieval memory was also affected by sub-chronic administration (6 mg/kg b.w. for 14 days) as well as higher single dose [9]. In another study, brivaracetam did not impact spatial memory in kainic acid-treated or sham rats when administered as 30 mg/kg i.p. one hour before the MWM test; however, in line with our results, the kainic acid-treated animals spent less time in the target quadrant on the probe day [11]. In contrast, Detrait et al. (2010) [10] showed that administering brivaracetam at single doses of 2.1, 6.8, or 21.0 mg/kg b.w. i.p. before each session did not alter spatial memory or swimming speed in either normal or amygdala-kindled rats. Also, brivaracetam did not affect long-term potentiation (LTP) induction in the CA1 hippocampal slices at concentrations that inhibited epileptiform activity (3–30 mM) [10]. Furthermore, 28-day brivaracetam administration (8.5 mg/kg b.w. i.p.) reduced spike-wave discharges linked to impairments in spatial memory and reversed memory dysfunction in a mouse model of Alzheimer’s disease [12].

The present study also investigated the impact of brivaracetam and ethanol on recognition memory using the NOR test. Three weeks of ethanol administration was found to disturb short-term recognition memory. Moreover, long-term memory was also negatively impacted, as all animals that received ethanol exhibited a lower discrimination index. In contrast to its effects on spatial memory, co-administration of brivaracetam with ethanol improved short-term memory without affecting long-term memory. Currently, no literature is available on the combined effects of brivaracetam and ethanol on recognition memory, with only a few reports focusing on the effects of the drug alone. Clinical trials suggest that brivaracetam has a favorable cognitive profile compared to other antiseizure medications. A study on 43 patients with epilepsy found short-term (after five days) and long-term brivaracetam use (i.e., after 25 weeks) to have beneficial effects on cognition, with improvements in attention and executive function, but no effect on verbal memory and reaction times [22]. However, during 12-month monotherapy with brivaracetam, one of the most frequent adverse effects was found to be memory disturbances (9.1%; *n* = 276), with memory problems being one of the leading causes of brivaracetam withdrawal (1.4%) [23]. In contrast, brivaracetam administered in two single doses had a negligible impact on neurocognitive functioning in healthy individuals [24].

While brivaracetam appears to have different effects on spatial and recognition memory, this should not necessarily be considered contradictory because different types of memory depend on distinct neural circuits. Spatial memory is strongly dependent on hippocampal function, as the hippocampus is essential for the formation and retrieval of spatial memory, which guides navigation and memory-based behavior [25]. Recognition memory relies on a distributed neuronal circuitry involving the perirhinal cortex, hippocampus, medial prefrontal cortex, and other interconnected cortical regions. The perirhinal cortex contributes to object recognition, whereas the hippocampus plays a role in object recognition memory, contributing to the encoding, consolidation, and retrieval of object-related information. Its involvement depends on the specific conditions of memory formation, including delay duration or contextual properties [26]. Both the hippocampus and cortex, like many other brain structures, widely express SV2A protein in their excitatory and inhibitory synapses [5]. Since hippocampal circuits rely on glutamatergic synaptic plasticity, the modulation of presynaptic neurotransmitter release through SV2A may differentially affect their function.

One of the fundamental cellular mechanisms underlying learning and memory is LTP, whose activity requires glutamatergic signaling mediated by NMDA and AMPA receptors. It has been found in previous studies that disrupting the induction or expression of LTP impairs learning and memory [27]. Hence, by dysregulating glutamatergic and GABAergic neurotransmission, ethanol may alter hippocampal LTP and synaptic plasticity mechanisms [28].

It has been demonstrated that mice with lower SV2A protein expression in the glutamatergic pyramidal neurons of the hippocampus show spatial memory deficit, thus supporting the importance of SV2A-dependent synaptic regulation for hippocampal cognitive function [29]. However, this animal model was developed in the context of epilepsy-related pathology, which limits direct comparison with the present study. Furthermore, Onwordi et al. (2025) report that SV2A, as a marker of synaptic terminal density, correlates positively with glutamatergic markers in the hippocampus of healthy volunteers, but not in patients with schizophrenia, suggesting that the relationship between SV2A and glutamatergic function may depend on the state of the neural network [30]. Unlike classical receptor agonists or antagonists, SV2A ligands such as brivaracetam modulate presynaptic neurotransmission by influencing synaptic vesicle cycling and neurotransmitter release rather than directly activating or blocking postsynaptic signaling pathways [31].

In PET studies of healthy volunteers, brivaracetam has been found to selectively bind to SV2A in the brain and produce dose-dependent SV2A occupancy [32]. However, no evidence currently suggests that the drug preferentially affects a specific brain region. Therefore, it is possible that differences in behavioral outcomes are more likely related to the functional characteristics of individual neural circuits and the pathophysiological state of the neural network rather than differences in regional brivaracetam action. As spatial memory formation strongly depends on hippocampal glutamatergic plasticity, hippocampal circuits may be particularly sensitive to changes in presynaptic neurotransmitter release associated with SV2A modulation. In contrast, recognition memory involves a broader network including the perirhinal cortex and prefrontal regions; as such, its behavioral performance may be differently affected by SV2A modulation, particularly in response to ethanol-induced alterations in neurotransmission. However, the precise mechanisms underlying this effect require further investigation.

In the present study, the rats taking part in the NOR test also underwent biochemical analyses, including neurotransmitter and oxidative stress parameter levels as well as the concentration of selected essential elements. Ethanol level did not significantly differ between ET and BRV + ET groups, which indicates that observed changes did not result from differences in ethanol intake. The GABA and glutamate concentrations were analyzed in three brain structures. As we have mentioned, different structures seem to be important in the formation of recognition memory, but the hippocampus is particularly important for the encoding and consolidation of contextual information [33]. Additionally, the cerebellum is involved in both motor and cognitive functions [34]. Ethanol affects GABAergic and glutamatergic transmission in a dose-dependent manner, with acute exposure increasing GABAergic transmission and decreasing glutamatergic transmission; in contrast, chronic ethanol intake may induce adaptive changes resulting in reduced GABAergic function and increased glutamatergic activity [35]. In the present study, all animals receiving ethanol only demonstrated higher GABA levels in the cerebellum than those that did not. This difference in the effect of ethanol on GABA or glutamate may be related to the duration of ethanol exposure in our experimental paradigm. Moreover, our present findings indicate that brivaracetam was associated with alterations in GABA and glutamate concentrations; however, no significant interaction effect was detected, indicating that this may only be a trend. All animals receiving brivaracetam demonstrated lower GABA concentrations in all studied brain structures than the non-drug rats. Moreover, increases in glutamate concentration were observed in the cerebellum and the cerebral cortex of all animals receiving the drug. In addition, brivaracetam administered alone significantly increased glutamate levels in the hippocampus.

Previous studies have found that brivaracetam does not directly modulate GABAergic or glutamatergic neurotransmission, and its pharmacological effects are primarily mediated through binding to SV2A. Brivaracetam has been shown to suppress astroglial L-glutamate release through SV2A-related mechanisms [7], and it was not found to have direct effects on ionotropic glutamate or GABA receptors in an electrophysiological study [8]. Given the established role of SV2A in regulating multiple stages of the synaptic vesicle cycle, including vesicle priming, calcium-dependent exocytosis, and neurotransmitter retention in synaptic vesicles [5], it is possible that the observed alterations in glutamate and GABA concentrations may result from prolonged modulation of SV2A-dependent synaptic vesicle function. An important consideration in the present analysis is that it is based on tissue neurotransmitter concentrations, which reflect the combined effects of neurotransmitter synthesis, vesicular storage, release, reuptake and metabolism. Therefore, higher glutamate/lower GABA concentration should not be interpreted as increased glutamatergic/GABAergic neurotransmission or/and activity. It cannot be excluded that alternative mechanisms, including altered neurotransmitter turnover and compensatory synaptic adaptations, may also play a part.

The observed tendency of brivaracetam to increase hippocampal glutamate levels suggests that glutamatergic neurotransmission may play a role in the improvement of short-term recognition memory observed in the NOR test. In a previous study, optogenetic inhibition of glutamatergic anterior CA2/CA3 terminals in anterior CA1 during the NOR test impaired object discrimination after a 5 min inter-trial interval, indicating that the pathway may be involved in retrieval of short-term object recognition memory [36]. However, in the present study, exploratory correlation analysis did not reveal any significant association between hippocampal glutamate levels and the discrimination index, indicating that the observed behavioral improvement cannot be directly attributed to glutamate concentration alone; moreover, brivaracetam did not improve long-term recognition memory, which may reflect distinct mechanistic requirements underlying different memory phases. Long-term memory formation requires additional mechanisms underlying consolidation and stabilization of memory traces, including activity-dependent gene expression, protein synthesis, and structural synaptic remodeling [37]. Therefore, ethanol-induced impairment of long-term memory may involve alterations in synaptic plasticity mechanisms that are not regulated by SV2A modulation alone.

As oxidative stress has been found to potentially impair memory and learning [38], the present study also examined the effect of administration on oxidative stress parameters, including MDA, SOD, and CAT in blood serum. As expected, MDA, one of the markers of oxidative damage, was increased in all animals receiving ethanol. This observation is in line with other studies indicating higher MDA levels to be associated with higher alcohol consumption, which causes increased lipid peroxidation and oxidative stress [14,39,40]. Furthermore, exploratory correlation analyses revealed that higher serum MDA levels were associated with poorer performance in both short- and long-term recognition memory in the NOR test. These correlations support the relationship between oxidative stress and cognitive alterations observed in the present study. In addition, brivaracetam administration was associated with reduced serum MDA levels, as all animals receiving brivaracetam exhibited lower MDA concentrations compared with non-treated animals. However, the absence of a significant ethanol × brivaracetam interaction indicates that brivaracetam did not specifically prevent ethanol-induced MDA elevation but rather exerted an overall effect on MDA levels. Moreover, as MDA was measured in serum, this relationship should not be interpreted as a direct indicator of oxidative damage within hippocampal circuits. Furthermore, all brivaracetam-treated rats demonstrated higher levels of SOD activity, which is part of the antioxidant defense. Recent findings in animals with induced ischemia indicate that a prior single dose of the drug (10 or 20 mg/kg b.w. given orally) dose-dependently increased both SOD and CAT activity compared to the untreated ischemic group [41]. In our study, an increase in CAT activity was noted following brivaracetam treatment; this is in line with a previous study where pentylenetetrazol-induced kindled rats were treated with brivaracetam (20 mg/kg b.w. i.p.) for 21 days [18]. While ethanol did not have a significant effect on SOD or CAT activities, a slight tendency, especially compared to controls, to decrease SOD and increase CAT was observed. In other studies, high levels of alcohol consumption have also been associated with lower SOD activity and variable impact on CAT activity, in a dose-dependent manner [14,40]. Furthermore, lower SOD activity was demonstrated in patients with Alzheimer’s disease, and a positive correlation was found between SOD and cognitive function [42]. Interestingly, despite the observed changes in SOD and CAT activities, these parameters were not associated with NOR discrimination indices. This lack of relationship may reflect the fact that antioxidant enzyme activities were measured in serum rather than in brain tissue.

Ethanol intake is known to influence the homeostasis of essential elements in the brain, mainly due to malnutrition and gastrointestinal problems. Excessive amounts or deficiency of particular ions may result in dysfunction of inter alia the central nervous system [43]. Trace elements play a role in maintaining redox balance as essential cofactors for enzymes. However, their deficiency or excess may be associated with oxidative damage and neurodegenerative diseases such as Alzheimer’s [44]. Our observations indicate that ethanol elevated manganese concentration in the brain, and this effect was reduced by brivaracetam. Furthermore, exploratory correlation analysis revealed that higher manganese concentrations were associated with poorer short- and long-term recognition memory in the NOR test. This suggests that altered manganese levels may be associated with cognitive alterations observed following ethanol exposure. Additionally, Nkpaa et al. (2019) reported that ethanol exacerbates manganese-induced spatial learning and memory deficits via oxidative stress and neuroinflammation in the rat hippocampus [45]. In another study, ethanol exposure also elevated manganese concentration in mice, and it was associated with upregulation of iron transporters; in addition, animals receiving ethanol demonstrated increased iron levels, but only at the highest dose of alcohol (10% *v*/*v*) [46].

Recent data indicate that moderate alcohol intake may be associated with higher iron levels in the brain of patients, suggesting that altered iron homeostasis may be involved in alcohol-related cognitive impairment [47]. In the present study, ethanol administration significantly increased iron concentrations compared with controls, as did brivaracetam treatment, alone and in combination with ethanol. These changes may reflect alterations in iron homeostasis following brivaracetam administration. However, it should also be noted that iron concentrations were not significantly correlated with NOR performance in the present study, and therefore further research is required to confirm any direct association with cognitive outcomes. Moreover, the present study did not include mechanistic assessments, such as iron transport pathways or ROS production, limiting the interpretation of the relationship between altered iron levels and oxidative damage.

Similarly, brivaracetam administration also resulted in increased copper concentrations. Elevated brain copper concentrations may cause cuproptosis, which has been associated with increased neuronal excitability, enhanced oxidative stress, mitochondrial dysfunction, and alterations in neurotransmitter release [48]. While the observed copper concentrations did not significantly correlate with NOR performance in the present study, such relationships may be revealed in future studies based on copper homeostasis and related regulatory mechanisms; these may also confirm whether copper concentrations are associated with functional alterations in copper metabolism. Nevertheless, any direct association between copper and cognitive outcomes remains uncertain.

Finally, our findings indicate elevated magnesium levels in the ethanol group, as well as in both brivaracetam-treated groups. Interestingly, magnesium concentration was negatively correlated with the long-term memory discrimination index, suggesting an association between altered magnesium concentrations and long-term cognitive performance. The observed alterations in iron, magnesium, and copper levels following brivaracetam administration warrant further investigation, as disturbances in their homeostasis have been implicated in oxidative and inflammatory processes [49].

## 4. Materials and Methods

### 4.1. Animals

Fifty-six male Wistar rats (242–315 g) were obtained from the Mossakowski Institute of Experimental and Clinical Medicine (Warsaw, Poland). The animals were housed in a temperature- and humidity-controlled room (20–22 °C, 55–60% relative humidity) with automatic 12 h light/dark cycles (lights on at 7:00 a.m.). All animals were allowed to acclimate to the conditions of the animal facility for one week before the start of the experiment. The animals were housed in groups of four in standard cages enriched with a plastic tunnel. Rats had free access to commercial chow. The experiments were conducted between 8:00 a.m. and 4:00 p.m. The animals were brought to the testing room 30 min before the start of each behavioral test. All experimental procedures complied with European Union Directive 2010/63/EU, ARRIVE guidelines, and were approved by the Local Ethical Committee for Experiments on Animals in Lodz, affiliated to the Medical University of Lodz, Poland (no. 9) (resolutions no. 66/ŁB/121/2018; 9/ŁB231/2022).

### 4.2. Drugs and Ethanol

**Figure 11:**
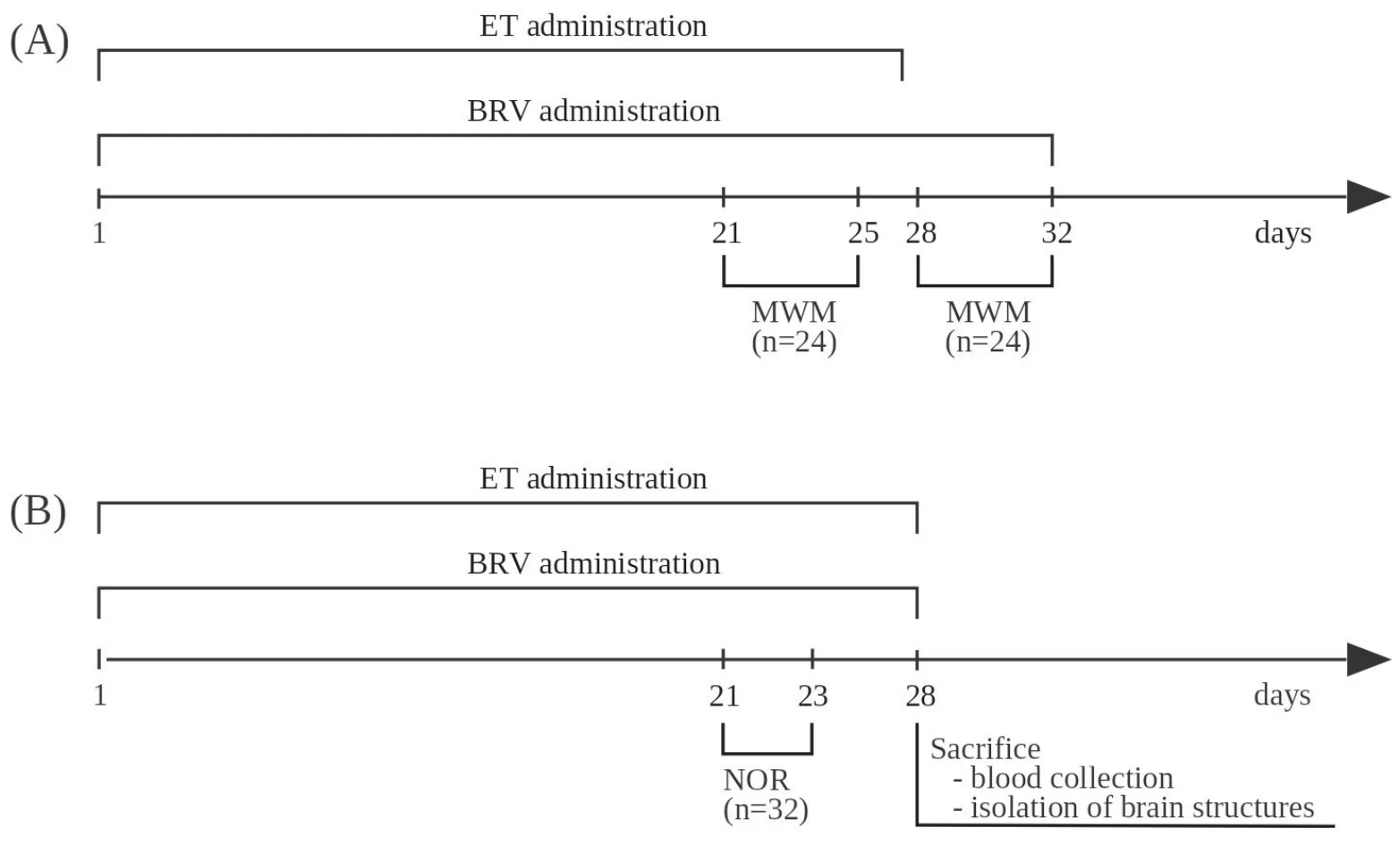
The timeline of experimental procedures. (**A**) MWM—Morris water maze test; (**B**) NOR—novel object recognition test. BRV—brivaracetam, ET—ethanol.

Initially, for the MWM, 24 rats were divided into three groups of eight animals: the brivaracetam and ethanol group (BRV + ET), the ethanol group (ET), and the control group (C). For the group assignment, all animals were weighed and ranked from lightest to heaviest and then assigned to groups in an equal manner based on their body mass. The other 32 rats were divided into four groups of eight animals, viz. brivaracetam (BRV), BRV + ET, ET, and C as described above. The rats participated in the NOR test and were subsequently sacrificed for further biochemical analysis. The timeline of experimental procedures is shown in Figure 11.

Brivaracetam was administered as a ready-made solution (Briviact^®^, 10 mg/mL solution; UCB Pharma, Brussels, Belgium) at a dose of 6 mg/kg b.w. (0.2 mL/100 g) once a day via an oral gavage directly into the stomach. The dose of the drug was adjusted based on previous findings [9]. Alcohol administration was conducted according to the forced ethanol intake model [50] with modifications according to Szmigielski [51]. This model was used to eliminate the risk of differential alcohol consumption among the groups. Appropriate alcohol concentrations were prepared from 95% food-grade ethanol by diluting it with water. Two groups (BRV + ET and ET) received 20% ethanol in two doses: 1.5 g/kg b.w. (0.75 mL/100 g; in the morning) and 3.5 g/kg b.w. (1.75 mL/100 g; in the afternoon) via oral gavage. Moreover, these animals had free access to 5% ethanol between 4:00 p.m. and 8:00 a.m., and water between 8:00 a.m. and 4:00 p.m. The other groups (C and BRV) had free access to water 24 h a day. The C group received 1% methylcellulose solution (0.2 mL/100 g). The drug, methylcellulose and ethanol were administered for 21 days before the start of the behavioral tests. During the behavioral tests, administration of these substances began after the testing was complete.

### 4.3. Behavioral Testing

#### 4.3.1. The MWM Test

The MWM is a well-established behavioral model used for the assessment of spatial memory and learning in rodents. The test protocol has been described in our previous study [9]. Briefly, the MWM consisted of a circular pool (180 cm diameter, 50 cm high) filled with 22 ±  2 °C water, and a submerged platform placed about 2 cm below the surface of the water in the selected quadrant. The pool was virtually divided into four quadrants and located in a dimly lit, quiet room with several extra-maze visual cues to facilitate navigation by the rat. Navigation parameters were analyzed using the ANY-maze software (ANY-maze 4.73. software, Wood Dale, IL, USA).

During the four-day session, acquisition tests evaluating spatial memory were conducted. For each daily trial, the rat was placed into the water facing the wall of the pool. After the rat found the platform, the trial was stopped, and the escape latency was recorded. The maximum trial length was 60 s. If the rat did not find the platform within this time, the experimenter guided the animal to the platform, and an escape latency of 60 s was recorded. The rats performed four trials a day, with a 60 s interval between them. The platform was positioned in the same place each time, but each trial started in a different quadrant. A decrease in the time needed to locate the platform was used as an indicator of functional spatial memory. Twenty-four hours after the last acquisition session, a probe trial without a hidden platform was conducted to assess the retrieval memory of the previous platform location. The animals were allowed to swim freely and search the pool for 60 s. If the retrieval memory was undisturbed, the rodents should spend more time swimming in the quadrant that had previously contained the hidden platform. The MWM test was conducted twice: first after 21 days of alcohol administration and then again during the period of alcohol discontinuation (see Figure 11).

#### 4.3.2. The NOR Test

Recognition memory was measured with the NOR test as described previously [9]. The NOR exploits the natural tendency of rodents to explore novel items. In the habituation phase (first day), the rats were put in an empty box for five minutes. The familiarization phase was conducted on the next day, and the animal was put back into the same box with two identical objects (A1 and A2) and allowed to explore them for three minutes. After a five-minute break, one of the familiar objects was replaced with a novel item (B), and short-term memory was measured during a three-minute trial. After 24 h, object B was replaced with a new, previously unknown object (C), and the animal was allowed to explore the environment for three minutes to assess long-term memory. Object exploration was defined as directing the snout at the object at no more than one cm distance, sniffing or touching the object with the nose. The exploratory movement of the rat was recorded with a video camera installed at the top of the box. Sample and test sessions were analyzed by two investigators independently and blinded to the treatment conditions, using self-developed software. The discrimination index (DI) was calculated using the following equation: (novel object exploration in seconds/both objects exploration in seconds) × 100%.

### 4.4. Biochemical Analysis

#### 4.4.1. Blood Ethanol Level Determination

The animals were euthanised by decapitation on Day 28 of the study, 30 min after receiving the last morning dose of ethanol (Figure 11). The whole-blood samples were collected for assays. The concentration of ethanol in the blood was measured enzymatically and expressed as g/L (‰).

#### 4.4.2. Assessment of GABA and Glutamate Levels in Selected Brain Structures

GABA and glutamate concentrations were assessed in three rat brain regions: cerebellum, hippocampus, and cerebral cortex, using the UPLC-FLD method, as previously described [52]. The concentrations of these neurotransmitters were expressed as µg/g wet tissue.

#### 4.4.3. Assessment of Serum Oxidative Stress Markers

The rat serum was obtained after centrifugation of whole blood. The material was used to determine the levels of the following oxidative stress markers according to the manufacturer’s instructions: TBARS (Thiobarbituric acid reactive substances, TBARS Assay Kit, Cayman Chemical, Ann Arbor, MI, USA, no. 700870), SOD (Superoxide Dismutase, Superoxide Dismutase Assay Kit, Cayman Chemical, Ann Arbor, MI, USA no. 706002) and CAT (Catalase, Catalase Assay Kit, Cayman Chemical, Ann Arbor, MI, USA no. 707002).

#### 4.4.4. Assessment of Selected Essential Elements Concentrations in the Brain

The concentrations of essential elements in the brain, including zinc, iron, magnesium, copper and manganese, were determined using flame atomic absorption spectrometry (FAAS) and graphite furnace atomic spectrometry (GFAAS). Instrumental conditions of both FAAS and GFAAS were investigated for each inorganic element. The brain samples were digested with ultrapure HNO_3_ and H_2_O_2_, using a microwave digestion system (MARSXpress, CEM Corporation, Matthews, NC, USA) [53]. The zinc, iron, and magnesium concentrations were determined using flame atomic absorption spectrometry on FAAS (Avanta GM; GBC Scientific Equipment Pty Ltd., Melbourne, Australia), while the levels of copper and manganese were measured using a Hitachi Z-8270 GFAAS (Hitachi, Ltd., Tokyo, Japan) with a Zeeman-type background correction, an autosampler, and a pyro-coated tube. Internal quality controls were used (Bovine liver 1577b (National Institute of Standards and Technology)) for each series of analyses. The repeatability error for 96% of the determinations did not exceed 10%. The detection limits (LOD) were as follows: zinc (0.01 μg/g wet tissue), iron (0.03 μg/g wet tissue), magnesium (0.3 μg/g wet tissue), copper (0.04 μg/g wet tissue), and manganese (0.06 μg/g wet tissue). The concentrations of essential elements in the brain were expressed as μg/g wet tissue.

### 4.5. Statistical Analysis

All data were processed using GraphPad Prism 10.6.0 (GraphPad, Boston, MA, USA). The data were tested for normality using the Shapiro–Wilk test and the homogeneity of variances was assessed using the Brown–Forsythe test. The obtained data were analyzed using either one-way ANOVA (factor: treatment) (probe trial MWM test), two-way ANOVA (factors: drug × ethanol) (neurotransmitter, oxidative stress markers and essential elements concentrations), and two-way RM ANOVA (two-way Repeated Measures ANOVA) (factors: time × treatment) (MWM test). When ANOVA indicated significant effects of factor(s) or interaction between them, it was followed by Tukey’s multiple comparisons test (two-way RM ANOVA), Sidak’s multiple comparisons test (one-way ANOVA or two-way ANOVA; significant interaction between factors) or an unpaired *t*-test (two-way ANOVA; significant effect of drug or ethanol). In all cases, *p* <  0.05 was considered significant. Effect sizes were reported alongside *p*-values for ANOVA results. Effect sizes were calculated as partial eta squared (ηp^2^) based on sums of squares obtained from the ANOVA tables. The data reported in this paper were presented as mean ±  standard error of the mean (SEM). Detailed descriptions of the tests are provided in Section 2 and figure captions. Additionally, exploratory Pearson’s correlation analyses were performed to assess the relationships between biochemical parameters and short- and long-term discrimination indices in the NOR test (*n* = 32). The strength of the correlation between variables is directly proportional to the magnitude of the correlation coefficient, indicating that a larger deviation from zero reflects a stronger correlation.

## 5. Conclusions

Brivaracetam produced distinct behavioral and biochemical effects. The drug was associated with alterations in spatial and retrieval memory during ethanol exposure and withdrawal. Ethanol-treated animals receiving brivaracetam showed improved short-term recognition memory without an effect on long-term recognition memory. Brivaracetam was also associated with alterations in GABA and glutamate concentrations in selected brain structures, although the functional significance of these changes remains unclear. The drug was associated with alterations in oxidative stress-related parameters, including reduced serum MDA levels and increased serum SOD activity in brivaracetam-treated animals, as well as increased serum CAT activity specifically in the brivaracetam group. Exploratory correlation analyses further revealed that higher MDA and manganese concentrations were associated with poorer recognition memory performance, which seems to confirm an association between oxidative status, elemental concentrations, and cognitive performance. Brivaracetam was associated with reduced manganese concentrations in ethanol-treated animals, whereas increases in iron, magnesium, and copper concentrations were observed following brivaracetam treatment. Further studies are required to clarify the mechanisms underlying the effects of brivaracetam.

### Limitations of the Study

The Morris water maze test was conducted on rats other than those used for biochemical analysis, as the MWM test was also performed during the alcohol discontinuation period. As a result, it is challenging to establish a direct correlation between observed behavioral outcomes and biochemical findings. The other limitation of the study is the lack of a brivaracetam-treated group in the Morris water maze test. The effects of the drug on memory have already been studied in our previous study [9], so we did not repeat this aspect. The study was performed exclusively in male rats. Therefore, potential sex-dependent differences cannot be excluded. In addition, no molecular validation was performed of the proposed mechanisms underlying the effects of brivaracetam or any pharmacokinetic assessment of brivaracetam; as such, the observed effects should be interpreted with caution. Oxidative stress parameters were measured in serum rather than directly in brain regions involved in cognition, which may not fully reflect region-specific brain alterations. Finally, the ethanol exposure model used in this study may not fully reproduce the complexity of long-term alcohol dependence.

## Data Availability

The original contributions presented in this study are included in the article. Further inquiries can be directed to the corresponding author.

## Abbreviations

The following abbreviations are used in this manuscript:

BRV	brivaracetam group
BRV + ET	brivaracetam and ethanol group
b.w.	body weight
C	control group
CAT	catalase
CB	cerebellum
CC	cerebral cortex
ET	ethanol group
GABA	gamma-aminobutyric acid
GPx	glutathione peroxidases
HP	hippocampus
i.g.	intragastric
i.p.	intraperitoneal
LTP	long-term potentiation
MDA	malondialdehyde
MWM	Morris Water Maze test
NOR	novel object recognition
RM ANOVA	Repeated Measures ANOVA
SOD	superoxide dismutase
SV2A	synaptic vesicle protein 2A
